# Effects of monoclonal antibody therapies on depression in Parkinson's disease and Alzheimer's disease: Systematic review and meta-analysis

**DOI:** 10.1177/13872877251378156

**Published:** 2025-09-19

**Authors:** Guy Gitlin-Leigh, Jack Wilson, Rebecca Howard, Robert Howard, Harry Costello

**Affiliations:** 1 Division of Psychiatry, University College London, London, UK; 2 Oxleas NHS Foundation Trust, Kent, UK

**Keywords:** Alzheimer's disease, depression, neurodegenerative diseases, neuroinflammation, Parkinson's disease, therapeutics/treatments

## Abstract

**Background:**

Depression in Alzheimer's disease (AD) and Parkinson's disease (PD) is common, disabling and difficult to treat. Pathological protein deposition in PD and AD has been associated with late-life depression, and inflammatory and vascular changes have been proposed as key mechanisms underlying depression in neurodegenerative disorders. Monoclonal antibody therapies (mAbs) effectively clear pathological protein aggregates in PD and AD but are associated with cerebral inflammation and microvascular damage.

**Objective:**

We conducted a systematic review and meta-analysis to determine whether mAb treatment influences the risk or severity of depression in AD and PD.

**Methods:**

Cochrane, Ovid MEDLINE/PubMed, PsycINFO and Embase databases were searched for articles published from inception to 8 April 2025. Randomized controlled trials of mAbs for PD or AD reporting a validated measure of depressive symptoms, or depression incidence were included and meta-analyzed.

**Results:**

We identified 13 studies including 8603 participants (treatment arm: n = 4690, placebo arm: n = 3913). All studies reported depression incidence as an adverse event, but none assessed changes in depressive symptom severity using standardized mood scales. Meta-analysis revealed no significant difference in the incidence of depression with mAb therapy (log risk ratio: −0.24, 95% CI (−0.52, 0.04), p = 0.09).

**Conclusions:**

We observed no significant association between mAb therapy and risk of depression in PD or AD. However, the absence of validated symptom assessments in these trials represents a critical gap in outcome reporting. Future trials should incorporate standardized depressive symptom measures as outcomes to evaluate the potential neuropsychiatric risks of these therapies.

## Introduction

Depression in Alzheimer's disease (AD) and Parkinson's disease (PD), the two most prevalent neurodegenerative disorders, is common, disabling and challenging to treat.^1–3^ Individuals with AD are twice as likely as age-matched controls to develop depression^2^ and up to one-third of PD patients will develop depression.^4^ Depression in both AD and PD contributes significantly to the disease burden and is associated with worse quality of life,^1,3,5^ exacerbates cognitive impairment,^6^ increases disability and has poor outcomes.^7^

Despite the widespread use of antidepressants for depression in AD, there is no evidence of therapeutic efficacy, and the limited number of trials in PD have produced conflicting results.^3,7^ Existing treatments for depression may be less effective in neurodegenerative conditions due to depression being a distinct syndrome related to pathological mechanisms specific to each disease.^7^ Substantial evidence supports that depression is an early prodromal symptom of AD and PD occurring up to 10 years prior to diagnosis and a consequence of distinct pathophysiology of each condition. For example biomarkers of AD pathology, such as tau and amyloid-β, are associated with the development of subsequent depression,^8^ and changes in α-synuclein metabolism (the major component of Lewy bodies in PD) is impaired in patients with depression.^9^

Over the last 15 years the drug development pipeline for both PD and AD has been dominated by trials of putatively disease-modifying monoclonal antibody therapies that target the clearance of misfolded protein aggregates implicated in the pathogenesis of these conditions, such as amyloid-β and α-synuclein.^10,11^ The mechanism of action of FDA approved monoclonal therapies such as lecanemab and donanemab relies on a neuroimmunological response induced by binding amyloid-β and stimulating microglial removal of amyloid plaques from the brain in people with AD.^12^ This can result in significant cerebral inflammation and vascular damage resulting in potentially life threatening microbleeds and oedema, referred to as amyloid-related imaging abnormalities (ARIA), that are symptomatic in up to a quarter of patients undergoing treatment.^13^

A consistent biological finding in depression in people who are not diagnosed with neurodegeneration is increased inflammation. Clinical studies of pro-inflammatory treatments such as interferon therapy in hepatitis C precipitates depression in a quarter of patients,^14^ and large longitudinal studies have found an association between inflammatory markers including C-reactive protein (CRP) and interleukin 6 (IL-6) with depression.^15^ Cerebrovascular changes in older adults have also been shown to predispose, precipitate and perpetuate depression.^16^

Although there have been mixed findings from trials of monoclonal antibody therapies on primary outcomes,^17,18^ and potentially questionable clinically meaningful effects,^19^ no study has investigated the effects of these therapies on mood symptoms or depression. This omission likely reflects the broader neglect of depression and other neuropsychiatric symptoms as outcomes in clinical trials—underscored by the limited pipeline of novel therapies targeting depression in neurodegenerative diseases, and the relative scarcity of high-quality trials in this area.^7^ If depression in neurodegenerative diseases such as PD and AD is a distinct syndrome driven by pathological protein aggregates such as amyloid-β and α-synuclein, then their clearance by monoclonal therapies may have antidepressant effects. Equally, these therapies could also potentially cause depression by triggering inflammatory and vascular mechanisms that may underlie the development of depressive symptoms.

To investigate this, we conducted the first systematic review and meta-analysis of the association between monoclonal antibody therapies and risk of depression in AD and PD.

## Methods

The following protocol was pre-registered on PROSPERO (number CRD42023216292).

### Systematic review

The Cochrane, Ovid MEDLINE/PubMed, PsycINFO and Embase databases were searched for articles published between 1 January 1946 and 8 April 2025 inclusive, with titles or abstracts containing the terms: (Alzheimer's OR dementia OR Parkinson's) AND (monoclonal antibod* OR immunotherapy OR anti-amyloid OR anti-Aβ OR anti-tau OR AAB-003 OR PF-05236812 OR Aducanumab OR BIIB037 OR BAN2401 OR mAb158 OR Bapineuzumab OR AAB-001 OR Crenezumab OR MABT5102A OR RG7412 OR Donanemab OR Lecanemab OR N3pG-Aβ OR LY3002813 OR GSK933776 OR Gantenerumab OR RO4909832 OR RG1450 OR LY2599666 OR LY3372993 OR MEDI1814 OR Ponezumab OR PF-04360365 OR SAR228810 OR Solanezumab OR LY2062430 OR UB-311 OR AN1792 OR AADvac1 OR ACI-35 OR Semorinemab OR Tilavonemab OR Zagotenemab OR Gosuranemab OR JNJ-63733657 OR E2814 OR Bepranemab OR Prasinezumab OR PRX002 AFFiRiS008 OR BIIB054).

Inclusion criteria were as follows: (1) Double blind randomized controlled trial (RCT) of a disease modifying monoclonal antibody therapy. (2) Trial included patients with a clinical diagnosis of AD or PD. (3) Reported validated measure of mood, depressive symptoms, or depression incidence (before and after treatment). (4) Published in English.

Articles were independently assessed for quality and risk of bias by GG and JW, using the Cochrane risk-of-bias tool for randomized trials (RoB 2) (see Supplemental Material). Any conflicts in quality assessment rating were resolved through in-person discussion.

### Meta-analysis

When three or more studies were identified, a meta-analysis was conducted to estimate the treatment effect on either the standardized mean difference in mood scores or the log risk ratio (log RR) of incident depression. Analyses were performed using the metafor package in R (version 4.4.3). A random-effects model was fitted with restricted maximum likelihood estimation to account for between-study heterogeneity.

Heterogeneity was analyzed using the approximate proportion of total variability (I^2^) where I² values of 25%, 50%, and 75% are typically considered low, moderate, and high heterogeneity, respectively. Leave-one-out sensitivity analysis using the leave1out() function from the *metafor* package was conducted to evaluate the influence of individual studies on the pooled effect estimate and to identify potentially influential outliers. For each iteration, one study was removed and the meta-analysis was re-run to evaluate changes in the estimated log risk ratio and its confidence interval.

Funnel plot asymmetry was assessed through both visual inspection and formal statistical tests. A funnel plot of study effect sizes against their standard errors was generated, and asymmetry was evaluated using Egger's regression test and Begg's rank correlation test. A two-sided p-value < 0.05 was considered statistically significant.

## Results

### Study characteristics

**Figure 1.**
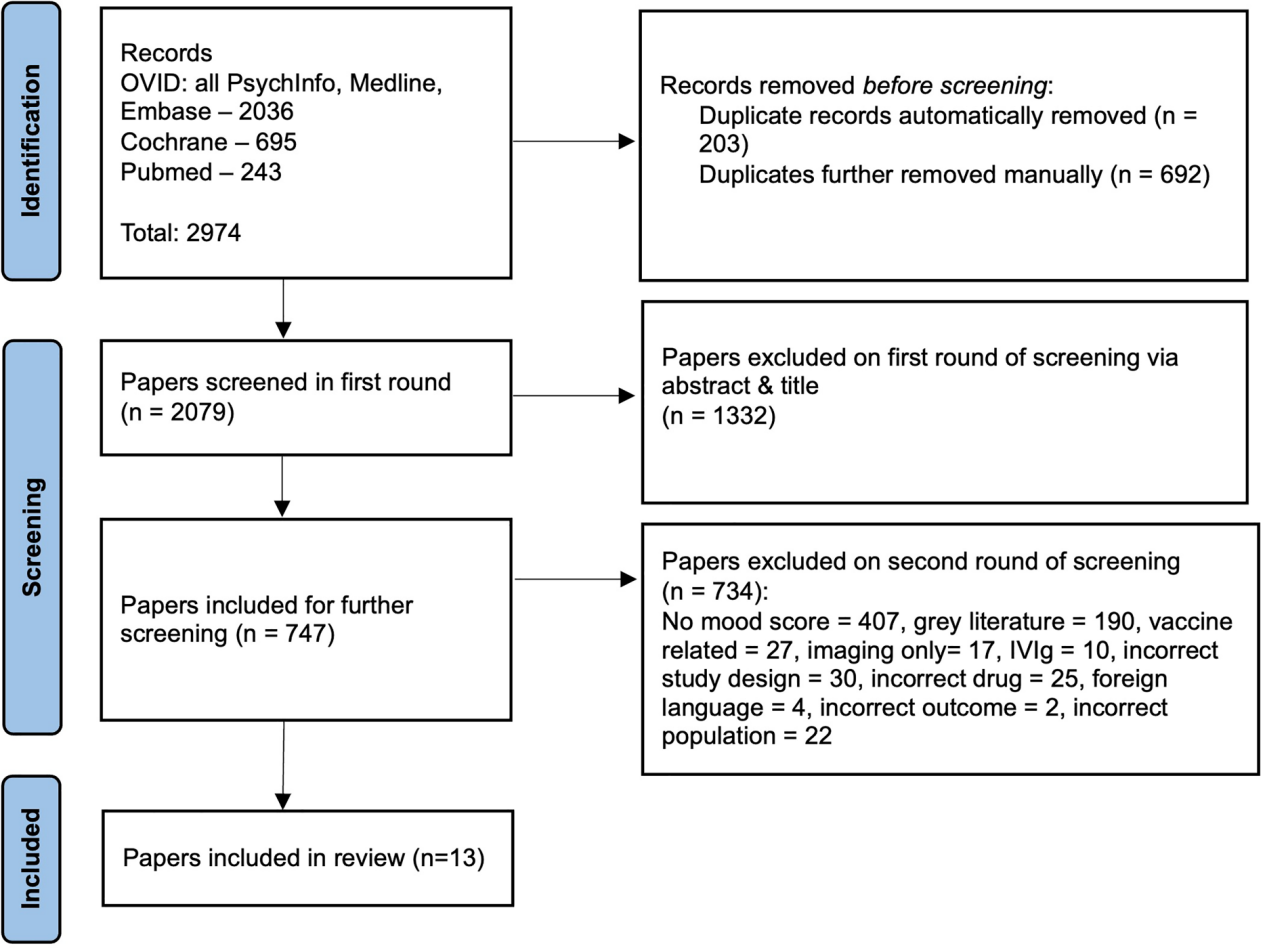
PRISMA flow diagram of study selection and inclusion.

Following duplicate removal, we identified 2079 studies; we excluded 1332 of these by title/abstract and retrieved the remaining 747 full papers (Figure 1). Data from 13 studies containing 8603 participants (treatment arm: n = 4690, placebo arm: n = 3913) were analyzed. The median number of patients per study was 256 (IQR 1294.5), mean participant age was 70.6 (SD 3.6) and mean duration of treatment was 88.9 weeks (SD 57.1) (see Table 1). The majority of studies (12/13) were monoclonal antibody trials for AD, all but one of which were therapies targeting amyloid-β (11/12). Only one included study was a trial of a monoclonal therapy (cinpanemab) for PD targeting α-synuclein.

**Table 1.** Summary of the main characteristics of included studies.

Study	Year	Drug	Country	AD/PD	N	Target	Duration (weeks)	AD/PD stage	Dose	Frequency	Mean age	ARIA with mAbs
Black et al.^20^	2010	Bapineuzumab	USA	AD	30	Amyloid-β	104	Mild/ moderate	0.5 mg/kg, 1.5 mg/kg, 5 mg/kg	once	72.9	-
Salloway et al.^21^	2014	Bapineuzumab	North America, Europe	AD	1121	Amyloid-β	78	Mild/moderate	0.5 mg/kg, 1.0 mg/kg	13 weekly	72.1	15.30%
EXPEDITION trials I & II^22^	2014	Solanezumab	North America, Europe, Australia	AD	2052	Amyloid-β	78	Mild/moderate	400 mg	4 weeks	72.5	ARIA E: 0.9%, ARIA H: 4.9%
Delnomdedieu et al.^23^	2016	Bapineuzumab	USA, Korea	AD	52	Amyloid-β	26	Mild/moderate	0.5, 1, 2, 4, & 8 mg/kg	13 weekly	67.8	ARIA E: 2.9%, ARIA H: 4.3%
Landen et al.^24^	2017	Ponezumab	30 sites globally	AD	194	Amyloid-β	104	Mild/moderate	Part a: 0.1 mg/kg, 0.5 mg/kg, 1 mg/kg, Part b: 3 mg/kg, 8.5 mg/kg	8 weeks	71.1	16.40%
Honig et al.^25^	2018	Solanezumab	North America, Europe, Australia	AD	2121	Amyloid-β	80	Mild	400 mg	4 weekly	72.7	-
Lu et al.^26^	2018	Bapineuzumab	USA	AD	40	Amyloid-β	20	Mild/moderate	5 mg, 10 mg, 40 mg, 80 mg	once	71.0	0%
BLAZE trial^27^	2018	Crenezumab	USA, Spain, France	AD	91	Amyloid-β	85	Mild/moderate	part 1: 300 mg, part 2: 15 mg/kg	part 1: 2 weekly, part 2: 4 weekly	69.4	ARIA E: 0%, ARIA H: 14.5%
Mintun et al.^28^	2021	Donanemab	USA, Canada	AD	256	Amyloid-β	76	Mild	700 mg for three doses then 1400 mg	4 weekly	75.2	ARIA E: 26.7%, ARIA-H: 8.4%
CREAD trials I & II^29^	2022	Crenezumab	Switzerland	AD	1611	Amyloid-β	100	Mild	60 mg/kg	4 weekly	70.8	ARIA H: 13.0%
Lang et al.^30^	2022	Cinpanemab	North America, Europe, Israel	PD	357	α-synuclein	52	Mild/moderate	250 mg, 1250 mg, or 3500 mg	4 weekly	60.1	na
Florian et al.^31^	2023	Tilavonemab	North America, Europe, Australia & New Zealand	AD	453	Tau	96	Mild	300 mg, 1000 mg, 2000mg	4 weekly	71.3	12.20%
Neve et al.^32^	2024	Gantenerumab	North America, Europe & Australia	AD	225	Amyloid-β	256	Mild	1200 mg	4 weekly	71.4	ARIA E: 26.2%, ARIA H: 18.2%

AD: Alzheimer's disease, PD: Parkinson's disease, - : missing, na: not applicable, ARIA H: Amyloid-related imaging abnormalities (microhemorrhages), ARIA E: Amyloid-related imaging abnormalities (oedema).

### Depression and other neuropsychiatric symptom outcomes

No studies of monoclonal antibody therapies for PD or AD reported change in depression symptom severity as an outcome. All included studies only reported depression incidence as an adverse event. Prevalence of depression at follow up across study arms was low (both arms = 5.9%, Tx = 5.5%, placebo = 6.4%). Most studies (8/13) excluded patients from participation in the trial if they had an existing diagnosis of depression or scored over a cut-off on a depression screening tool (3/13). Consequently, most studies reported the emergence of a new diagnosis of depression following initiation of treatment. Overall neuropsychiatric symptom burden was measured using the Neuropsychiatric Inventory questionnaire (NPI), by 4/12 studies, all of which reported no significant change in NPI score between treatment and placebo groups at follow up.

### Meta-analysis and sensitivity analyses

**Figure 2.**
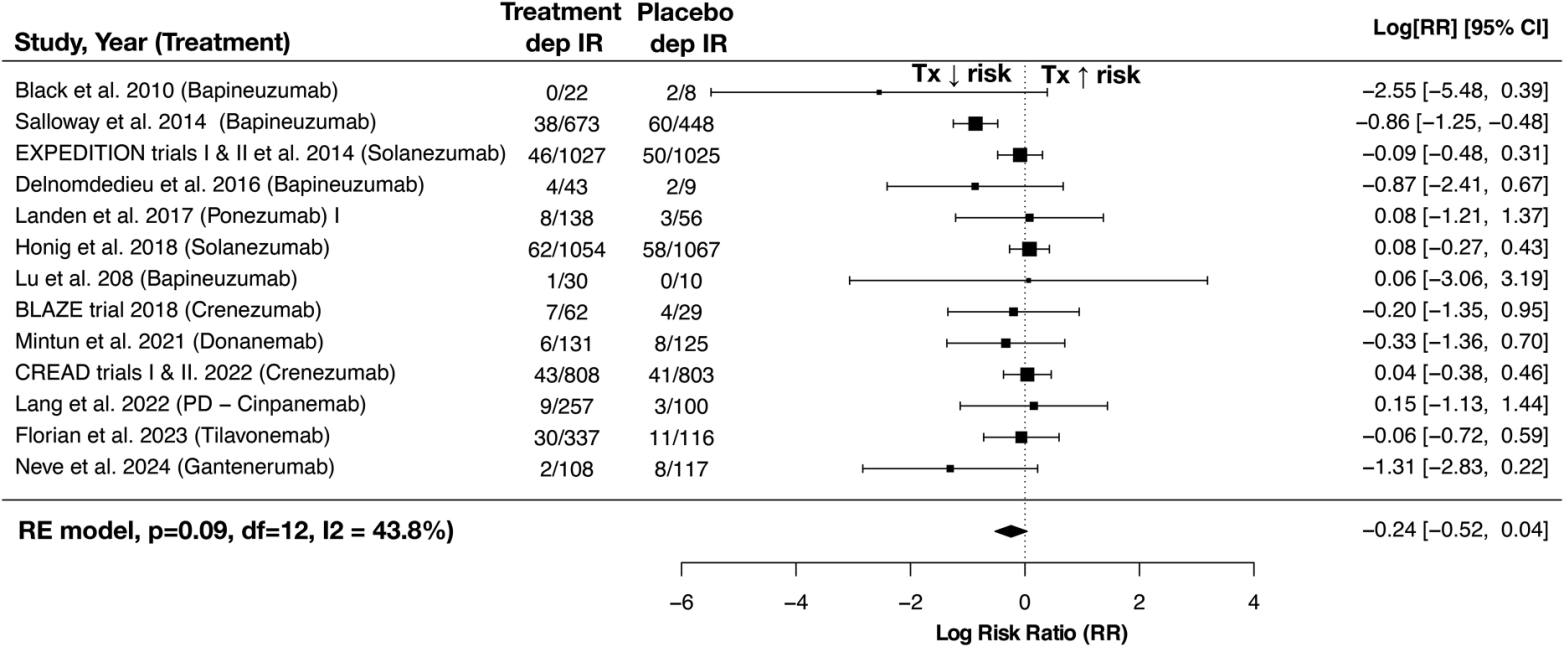
Forest plot of meta-analysis of risk ratios for depression occurring as an adverse event. RE model: random-effects model, p: p-value, df: degrees of freedom, IR: incidence rate, Tx: treatment.

**Figure 3.**
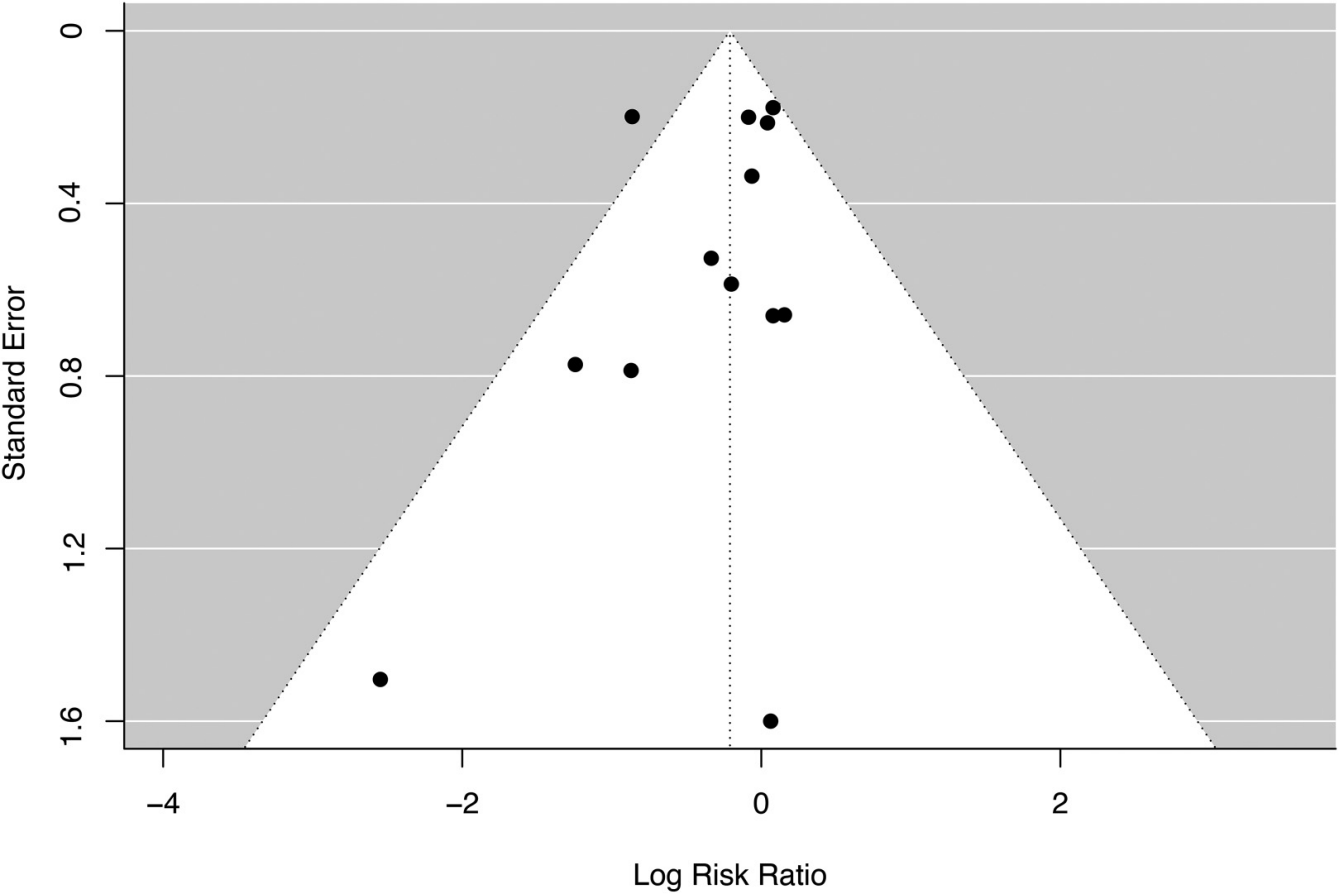
Funnel plot analysis of included studies publication bias.

Meta-analysis across all trials revealed no significant relationship between monoclonal antibody therapy and risk of developing depression (log risk ratio: −0.24, 95% CI (−0.52, 0.04), p = 0.09) (Figure 2). In a subgroup analysis limited to therapies targeting amyloid-β, no significant relationship was found between treatment and depression risk in AD (log risk ratio: −0.29, 95% CI (−0.61, 0.03), p = 0.08). Leave-one-out analysis found that no single study had a significant or disproportionate influence on the direction or overall size of effect (see Supplemental Figure 1 for leave-one-out sensitivity analysis). Overall interstudy heterogeneity was moderate (I^2^ = 43.8%), and likely a consequence of variability in trial duration and population characteristics (see Table 1 for summary of these differences). Funnel plot asymmetry showed no strong evidence to support publication bias. Statistical analysis using Egger's test did not indicate significant evidence of small-study effects (t = –0.82, df = 11, p = 0.43), with an intercept estimate of −0.07 (95% CI: −0.53 to 0.40). Begg's test showed a non-significant but borderline rank correlation between effect estimates and their standard errors (Kendall's tau = –0.38, p = 0.076). These findings were consistent with visual inspection of the funnel plot (see Figure 3).

### Risk of bias

Quality assessment using a modified RoB-2, found that the overall risk of bias varied across studies (see Table 2). Overall judgement of bias for the majority of studies (11/13) met criteria for “some concerns”, particularly in the domains of outcome measurement and reported results. This included several studies that did not report how clinical depression was monitored for or diagnosed as an outcome, or reported ‘depressive symptoms’ with no qualifying statement indicating how this was assessed and measured. All included studies were industry-sponsored and funded by the pharmaceutical sponsor of the monoclonal antibody. Most studies matched groups at baseline, reported effective randomization procedures, accounted for missing outcome data and reported comprehensive data on participant retention and drop-outs. However, some studies did not adequately report blinding procedures or adherence to the intervention protocol, resulting in “some concerns” (for further detailed rating scores see Supplemental Table 1).

**Table 2.** Quality assessment and summary risk of bias rating for each Cochrane risk-of-bias tool for randomized trials domain.

Study	Risk of bias (RoB-2) domain rating				
	Randomization process	Deviations from intended interventions	Missing outcome data	Outcome measurement	Selection of reported results
Black et al. (2010)^20^	Low	Some concerns	Low	Some concerns	Some concerns
Salloway et al. (2014)^21^	Low	Some concerns	High	Low	Some concerns
EXPEDITION trials I & II (2014)^22^	Low	Low	Some concerns	Low	Some concerns
Delnomdedieu et al. (2016)^23^	Low	Low	Low	Some concerns	Some concerns
Landen et al. (2017)^24^	Low	Low	Low	Low	Low
Honig et al. (2018)^25^	Low	Low	Low	Some concerns	Low
Lu et al. (2018)^26^	Low	Low	Low	High	Some concerns
BLAZE trial (2018)^27^	Low	Some concerns	Low	Some concerns	Low
Mintun et al. (2021)^28^	Low	Some concerns	Low	Some concerns	Some concerns
CREAD trials I & II (2022)^29^	Low	Some concerns	Low	Some concerns	Some concerns
Lang et al. (2022)^30^	Low	Low	Low	Some concerns	Some concerns
Florian et al. (2023)^31^	Low	Low	Low	Low	Some concerns
Neve et al. (2024)^32^	Low	Some concerns	Low	Some concerns	Some concerns

## Discussion

We conducted the first systematic review and meta-analysis to investigate the effects of monoclonal antibody therapies on mood symptoms and depression in AD and PD. Our findings suggest there is no increased risk of depression associated with these therapies. However, we identified only 13 studies that recorded depression as an outcome and none that reported change in depressive symptom severity. Since 2005, over a hundred trials have been registered investigating monoclonal antibodies in patients with AD or PD, indicating only a small minority of trials measure or report mood symptoms or depression as an outcome measure.

Depressive symptoms in AD and PD are intrinsically related to the core pathology of these conditions, are often the earliest symptoms experienced by patients and can be highly disabling. Lack of measurement and reporting of the effects of monoclonal therapies on depression and other psychiatric symptoms in AD and PD, potentially increases the risk for missed prevention of future patient harm and neglects an opportunity to take advantage of the greatest driver of drug development in Psychiatry - serendipity. In the examples of levetiracetam for epilepsy and interferon-alpha therapy for hepatitis C, in which psychiatric adverse events occur in up to 16% and 25% of patients respectively,^33,34^ it took several years following licensing and marketing of these therapies before these risks were recognized. This was in part due to the lack of measurement and reporting in the pivotal licensing trials. In contrast, several monoclonal antibody therapies used to treat inflammatory disorders have been associated with improvement in depressive symptoms and are now informing future trials of these therapies for treatment resistant depression.^35,36^

Our findings have potential implications for understanding the mechanisms underlying depression in AD and PD.^16,36^ Vascular and inflammatory processes have been proposed as key mechanisms underlying the emergence of depression.^37–39^ Monoclonal amyloid-β therapies induce widespread cerebral inflammation, oedema and microhemorrhages that manifest as ARIA early during treatment and are related to dose, existing vascular changes and Apolipoprotein E (*APOE*) genotype.^13^ Prevalence of ARIA reported in the included studies in our review ranged from 0–26.7%, but asymptomatic vascular or inflammatory changes that are not detectable via conventional MRI are likely to be present in the majority of patients receiving treatment. Consequently, we would anticipate that, if vascular and inflammatory mechanisms are key to the emergence of depression, treatment with amyloid-β therapies would lead to increases in incidence of depression. Our finding that this is not the case indicates that vascular and inflammatory processes may not be key causal mechanisms underlying depression in PD and AD, or that the specific inflammatory and vascular processes associated with depression are not triggered or may even be ameliorated by these treatments.

Previous studies have shown that depression in older adults is associated with prodromal AD pathology including accumulation of amyloid-β and tau deposits.^8,40^ All monoclonal therapies included in our analysis were shown to effectively clear their target proteins in the brain, as measured using PET imaging. With effective clearance, a reduction in incidence of depression driven by amyloid or tau pathology could be predicted but was not observed. Although this could be interpreted as evidence against pathological protein deposition driving depression in AD and PD, it is more likely a consequence of the highly selected participant groups recruited by included studies.

A limitation to the interpretation of our results is that most trials excluded patients with a diagnosis of depression. Given that depression is among the earliest and most common neuropsychiatric symptoms in AD and PD, affecting at least one-third of patients, this exclusion reduces the generalizability of trial populations.^41,42^ Excluding these patients not only leads to an unrepresentative patient group within trials, but also limits insight into how these treatments might affect individuals with heightened vulnerability to depression or elevated baseline symptom severity. Nevertheless, the average follow-up duration across included studies exceeded one year, providing sufficient time to detect differences in the incidence of new-onset depression.

Differences in monoclonal antibody dose, administration frequency, and treatment duration across trials may have influenced depression-related outcomes and contributed to between-study heterogeneity. However, the limited number of studies and variability in reporting prevented further subgroup analyses.

Only one included trial was conducted in PD, and the majority of studies investigated anti-amyloid monoclonal antibody therapies, limiting the generalizability of findings to PD and to other protein targets. Although fewer monoclonal antibody trials have been conducted in PD compared to AD, several PD trials were identified in our initial search. Most of these studies lacked any measurement of depression as an outcome. Therefore, the limited representation of PD in our review reflects both the relative scarcity of PD mAb trials and the absence of relevant outcome reporting, rather than the application of restrictive eligibility criteria.

Finally, all included studies were industry-sponsored, which may increase the risk of selective outcome reporting, particularly with respect to adverse events. While no evidence of publication bias was detected in funnel plot analyses or formal tests for asymmetry, the potential for reporting bias cannot be fully excluded.

### Conclusion

We conducted the first systematic review and meta-analysis examining the relationship between monoclonal antibody therapy and the risk of depression in PD and AD. We observed no significant association of monoclonal antibody therapies on risk of depression in either condition. However, no study reported change in depressive symptom severity and a small minority of all monoclonal trials to date have screened for depression as an adverse event.

Future trials of potentially disease modifying therapies in PD and AD should incorporate validated depression symptom measures as key secondary outcomes to identify the potential neuropsychiatric risks and therapeutic benefits of these therapies.

## Supplemental Material

sj-docx-1-alz-10.1177_13872877251378156 - Supplemental material for Effects of monoclonal antibody therapies on depression in Parkinson's disease and Alzheimer's disease: Systematic review and meta-analysis Supplemental material, sj-docx-1-alz-10.1177_13872877251378156 for Effects of monoclonal antibody therapies on depression in Parkinson's disease and Alzheimer's disease: Systematic review and meta-analysis by Guy Gitlin-Leigh, Jack Wilson, Rebecca Howard, Robert Howard and Harry Costello in Journal of Alzheimer's Disease
