# Supplementary material for: Effects of monoclonal antibody therapies on depression in Parkinson's disease and Alzheimer's disease: Systematic review and meta-analysis
Source: J Alzheimers Dis. 2025 Sep 19;108(2):500–8. doi: 10.1177/13872877251378156 (PMC13187836; doi:10.1177/13872877251378156)
Supplement: sj-docx-1-alz-10.1177_13872877251378156 - Supplemental material for Effects of monoclonal antibody therapies on depression in Parkinson's disease and Alzheimer's disease: Systematic review and meta-analysis [file sj-docx-1-alz-10.1177_13872877251378156.docx]

**Supplemental Material**

**Effects of monoclonal antibody therapies on depression in Parkinson’s disease and Alzheimer’s disease: Systematic review and meta-analysis**

**Revised Cochrane risk-o**^1^**f-bias tool for randomized trials (RoB 2) items:**

***(Y/PY = ‘Yes’ or ‘Probably yes’; N/PN = ‘No’ or ‘Probably no’; NI = ‘No information’)***

**Domain 1: Risk of bias arising from the randomization process**

1.1 Was the allocation sequence random?

1.2 Was the allocation sequence concealed until participants were enrolled and assigned to interventions?

1.3 Did baseline differences between intervention groups suggest a problem with the randomization process?

Overall judgement Domain 1 - Low / High / Some concerns

**Domain 2: Risk of bias due to deviations from the intended interventions**

2.1. Were participants aware of their assigned intervention during the trial?

2.2. Were carers and people delivering the interventions aware of participants assigned intervention during the trial?

2.3. If Y/PY/NI to 2.1 or 2.2: Were there deviations from the intended intervention that arose because of the trial context?

2.4 If Y/PY to 2.3: Were these deviations likely to have affected the outcome?

2.5. If Y/PY/NI to 2.4: Were these deviations from intended intervention balanced between groups?

2.6 Was an appropriate analysis used to estimate the effect of assignment to intervention?

2.7 If N/PN/NI to 2.6: Was there potential for a substantial impact (on the result) of the failure to analyse participants in the group to which they were randomized?

Overall judgement Domain 2 - Low / High / Some concerns

**Domain 3: Missing outcome data**

3.1 Were data for this outcome available for all, or nearly all, participants randomized?

3.2 If N/PN/NI to 3.1: Is there evidence that the result was not biased by missing outcome data?

3.3 If N/PN to 3.2: Could missingness in the outcome depend on its true value?

3.4 If Y/PY/NI to 3.3: Is it likely that missingness in the outcome depended on its true value?

Overall judgement Domain 3 - Low / High / Some concerns

**Domain 4: Risk of bias in measurement of the outcome**

4.1 Was the method of measuring the outcome inappropriate?

4.2 Could measurement or ascertainment of the outcome have differed between intervention groups?

4.3 If N/PN/NI to 4.1 and 4.2: Were outcome assessors aware of the intervention received by study participants?

4.4 If Y/PY/NI to 4.3: Could assessment of the outcome have been influenced by knowledge of intervention received?

4.5 If Y/PY/NI to 4.4: Is it likely that assessment of the outcome was influenced by knowledge of intervention received?

Overall judgement Domain 3 - Low / High / Some concerns

**Domain 5: Risk of bias in selection of the reported result**

5.1 Were the data that produced this result analysed in accordance with a pre-specified

analysis plan that was finalized before unblinded outcome data were available for analysis?

Is the numerical result being assessed likely to have been selected, on the basis of the

results, from...

5.2. ... multiple eligible outcome measurements (e.g. scales, definitions, time points) within the outcome domain?

5.3 ... multiple eligible analyses of the data?

Overall judgement Domain 3 - Low / High / Some concerns

**Included study references**

1. Black RS, Sperling RA, Safirstein B, et al. A single ascending dose study of bapineuzumab in patients with Alzheimer disease. *Alzheimer Dis Assoc Disord* 2010; 24: 198-203.
2. Salloway S, Sperling R, Fox NC, et al. Two phase 3 trials of bapineuzumab in mild-to-moderate Alzheimer’s disease. *N Engl J Med* 2014; 370: 322–333.
3. Doody RS, Thomas RG, Farlow M, et al. Phase 3 trials of solanezumab for mild-to-moderate Alzheimer’s disease. *N Engl J Med* 2014; 370: 311–321.
4. Delnomdedieu M, Duvvuri S, Li DJ, et al. First-In-Human safety and long-term exposure data for AAB-003 (PF-05236812) and biomarkers after intravenous infusions of escalating doses in patients with mild to moderate Alzheimer’s disease. *Alzheimers Res Ther* 2016; 8: 12.
5. Landen JW, Andreasen N, Cronenberger CL, et al. Ponezumab in mild-to-moderate Alzheimer’s disease: Randomized phase II PET-PIB study. *Alzheimers Dement (N Y)* 2017; 3: 393–401.
6. Honig LS, Vellas B, Woodward M, et al. Trial of solanezumab for mild dementia due to Alzheimer’s disease. *N Engl J Med* 2018; 378: 321–330.
7. Lu M and Brashear HR. Pharmacokinetics, pharmacodynamics, and safety of subcutaneous bapineuzumab: a single-ascending-dose study in patients with mild to moderate Alzheimer disease. *Clin Pharmacol Drug Dev* 2019; 8: 326–335.
8. Salloway S, Honigberg LA, Cho W, et al. Amyloid positron emission tomography and cerebrospinal fluid results from a crenezumab anti-amyloid-beta antibody double-blind, placebo-controlled, randomized phase II study in mild-to-moderate Alzheimer’s disease (BLAZE). *Alzheimers Res Ther* 2018; 10: 96.
9. Mintun MA, Lo AC, Evans CD, et al. Donanemab in early Alzheimer’s disease. *N Engl J Med* 2021; 384: 1691–1704.
10. Ostrowitzki S, Bittner T, Sink KM, et al. Evaluating the safety and efficacy of crenezumab vs placebo in adults with early Alzheimer disease: two phase 3 randomized placebo-controlled trials. *JAMA Neurol* 2022; 79: 1113–1121.
11. Lang AE, Siderowf AD, Macklin EA, et al. Trial of cinpanemab in early Parkinson’s disease. *N Engl J Med* 2022; 387: 408–420.
12. Florian H, Wang D, Arnold SE, et al. Tilavonemab in early Alzheimer’s disease: results from a phase 2, randomized, double-blind study. *Brain* 2023; 146: 2275–2284.
13. Neve A, Das B, Wojtowicz J, et al. Long-term safety of gantenerumab in participants with Alzheimer’s disease: a phase III, double-blind, and open-label extension study (Marguerite RoAD). *J Alzheimers Dis* 2024; 101: 353–367.

**Supplement Figure 1.** Leave-one-out sensitivity analysis

**Supplemental Table 1.** Revised Cochrane risk-of-bias tool ratings for included studies. *(Y/PY = ‘Yes’ or ‘Probably yes’; N/PN = ‘No’ or ‘Probably no’; NI = ‘No information’)*

| **Risk of bias due to:** | **Randomization process** | | | | **Deviations from the intended interventions** | | | | | | | | **Missing outcome data** | | | | | **Measurement of the outcome** | | | | | | **Selection of the reported result** | | | |
| --- | --- | --- | --- | --- | --- | --- | --- | --- | --- | --- | --- | --- | --- | --- | --- | --- | --- | --- | --- | --- | --- | --- | --- | --- | --- | --- | --- |
| **Study** | 1.1 | 1.2 | 1.3 | 1 overall | 2.1 | 2.2 | 2.3 | 2.4. | 2.5 | 2.6 | 2.7 | 2 overall | 3.1 | 3.2 | 3.3 | 3.4 | 3 overall | 4.1 | 4.2 | 4.3 | 4.4 | 4.5 | 4 overall | 5.1 | 5.2 | 5.3 | 5 overall |
| Black et al. (2010) | PY | PY | N | Low | PN | PN | NI | NI | Y | PN | PN | Some concerns | Y | NI | PY | PY | Low | NI | NI | NI | NI | PN | Some concerns | PN | NI | PN | Some concerns |
| Salloway et al. (2014) | PY | PY | N | Low | PY | PY | PN | N | NI | Y | Y | Some concerns | N | N | Y | Y | High | N | N | PN | PY | PN | Low | NI | PY | PN | Some concerns |
| EXPEDITION trials I & II (2014) | PY | PY | N | Low | PN | PN | N | NI | Y | Y | NA | Low | N | PN | PY | PY | Some concerns | NI | PN | PN | NI | NI | Low | PY | NI | PN | Some concerns |
| Delnomdedieu et al. (2016) | PY | PY | PN | Low | PN | PN | NI | NI | NI | PY | NI | Low | Y | NI | NI | NI | Low | NI | NI | PN | NI | NI | Some concerns | PY | NI | PN | Some concerns |
| Landen et al. (2017) | PY | PY | N | Low | PY | PY | PN | NI | NI | PY | NI | Low | Y | NI | NI | NI | Low | Y | Y | PN | NI | NI | Low | PY | NI | PN | Low |
| Honig et al. (2018) | Y | Y | PN | Low | PN | PN | NI | NI | NI | PY | NI | Low | PN | NI | NI | NI | Low | NI | NI | PN | NI | NI | Some concerns | PY | PN | PN | Low |
| Lu et al. (2018) | PY | PY | PN | Low | PY | PY | PN | NI | NI | Y | NI | Low | Y | NI | PY | NI | Low | PY | PN | NI | NI | NI | High | PY | NI | PN | Some concerns |
| BLAZE trial (2018) | Y | Y | N | Low | PN | PY | PY | PN | NI | N | PN | Some concerns | Y | N | PN | N | Low | NI | NI | PN | PN | PN | Some concerns | PY | NI | NI | Low |
| Mintun et al. (2021) | Y | PY | N | Low | N | NI | NI | N | NI | PN | PN | Some concerns | N | N | N | NI | Low | NI | PN | NI | NI | NI | Some concerns | NI | NI | NI | Some concerns |
| CREAD trials I & II (2022) | PY | PY | N | Low | PN | PN | NI | NI | NI | PY | NI | Some concerns | Y | PY | NI | NI | Low | NI | NI | PY | NI | NI | Some concerns | PY | NI | PN | Some concerns |
| Lang et al. (2022) | PY | PY | N | Low | PN | PN | Y | N | Y | PY | N | Low | PY | Y | N | N | Low | NI | NI | NI | NI | PN | Some concerns | PY | NI | PN | Some concerns |
| Florian et al. (2023) | PY | PY | N | Low | PN | PN | NA | NA | NA | Y | NA | Low | PY | PN | NI | NI | Low | N | PN | PN | PN | PN | Low | PY | NI | PN | Some concerns |
| Neve et al. (2024) | PY | PY | N | Low | Y | y | PN | N | PY | PY | N | Some concerns | PN | PN | PY | N | Some concerns | N | PN | Y | PY | PY | Some concerns | PN | NI | PN | Some concerns |
